# Cell Death, by Any Other Name…

**DOI:** 10.3390/cells13040325

**Published:** 2024-02-10

**Authors:** Mustapha Kandouz

**Affiliations:** 1 Department of Pathology, School of Medicine, Wayne State University, 540 East Canfield Avenue, Detroit, MI 48201, USA; ag1764@wayne.edu; 2 Karmanos Cancer Institute, Wayne State University, Detroit, MI 48201, USA

**Keywords:** apoptosis, necrosis, autophagy, necroptosis, ferroptosis, pyroptosis, lysosome-dependent cell death, bystander effect, intercellular communication, ephrins

## Abstract

Studies trying to understand cell death, this ultimate biological process, can be traced back to a century ago. Yet, unlike many other fashionable research interests, research on cell death is more alive than ever. New modes of cell death are discovered in specific contexts, as are new molecular pathways. But what is “cell death”, really? This question has not found a definitive answer yet. Nevertheless, part of the answer is irreversibility, whereby cells can no longer recover from stress or injury. Here, we identify the most distinctive features of different modes of cell death, focusing on the executive final stages. In addition to the final stages, these modes can differ in their triggering stimulus, thus referring to the initial stages. Within this framework, we use a few illustrative examples to examine how intercellular communication factors in the demise of cells. First, we discuss the interplay between cell–cell communication and cell death during a few steps in the early development of multicellular organisms. Next, we will discuss this interplay in a fully developed and functional tissue, the gut, which is among the most rapidly renewing tissues in the body and, therefore, makes extensive use of cell death. Furthermore, we will discuss how the balance between cell death and communication is modified during a pathological condition, i.e., colon tumorigenesis, and how it could shed light on resistance to cancer therapy. Finally, we briefly review data on the role of cell–cell communication modes in the propagation of cell death signals and how this has been considered as a potential therapeutic approach. Far from vainly trying to provide a comprehensive review, we launch an invitation to ponder over the significance of cell death diversity and how it provides multiple opportunities for the contribution of various modes of intercellular communication.

## 1. Introduction: The Dead Cell

It is now customary in the field of cell death to trace back its earliest mentions to more than a century ago, in fact, to the 19th century German naturalist Carl Vogt and his work on the nervous system of toad embryos or even to earlier observations [1]. Many decades later, histological studies of ischemic liver injury carried out between 1962 and 1964 were behind the distinction between classical necrotic cell death and what will later be known as apoptotic cell death or simply apoptosis [2].

Although every type of cell death proceeds through different stages, it is when it has reached the point of no return, where the decay inflicted to it is irreversible, that a cell would be considered dead. A group of leading researchers in the field, part of the “Nomenclature Committee on Cell Death”, has proposed a set of molecular and morphological criteria to identify a “dead cell” [3]. So, apart from initiatory and regulatory mechanisms, what exactly are these landmark morphological and biochemical events that define the essence of cell death? We know that cells can recover from apoptosis up to a certain point [4,5,6,7,8,9,10,11], and a name has been coined for that mechanism of recovery: anastasis, from the Greek “rising to life”, also known as “resurrection” [5]. While saving cells from certain death is a desirable outcome in many situations, particularly in degenerative diseases, it might come at a highly dangerous price, as it allows cells which have acquired mutations to undergo oncogenic transformation [12,13,14]. A profiling study suggested that, even during apoptosis, cells express a “poise for recovery” set of genes encoding survival proteins that, although not translated, can help cells to recover if the apoptotic stress is removed [15]. In fact, cells have a “life”, at least biochemically speaking, after death [16], and some processes persist even after the cell’s remnants have been engulfed by phagocytosis, as is the case with the release of lysosomal cathepsins into the cytosol [17,18]. The generated apoptotic bodies (ABs) even acquire a second identity as intercellular communication moieties, similar to exosomes and other extracellular vesicles [19,20,21]. The challenge regarding any event occurring beyond the point where anastasis is impossible is to know whether it is a consequence of death or an active executioner of it. However, a more pressing aim for us here is to have an idea about the events surrounding the border between the life and death of the cell.

**Figure 1:**
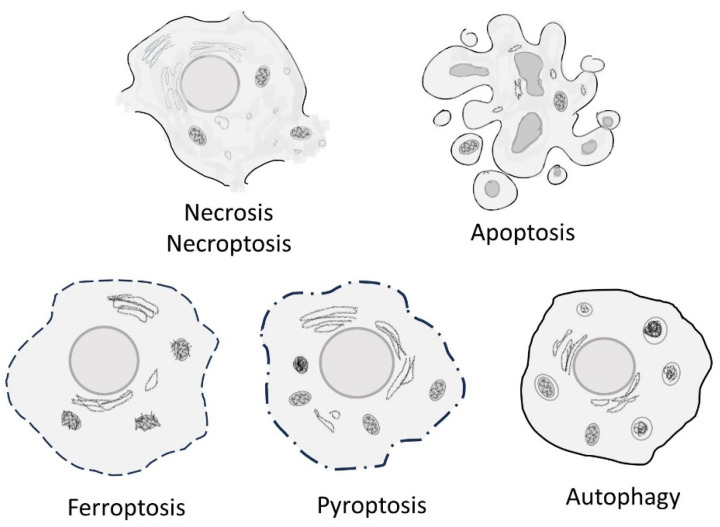
The boundaries between different modes of cell death are not clear-cut, as one often leads to the other, depends on it, or at least shares common features with it.

The accidental death of cells known as necrosis (from the Greek etymology *nekrōsis*, “*nekros*” meaning (dead) corpse and “*-osis*” meaning a process or condition) appears as the swelling and rupture of the organelles and cell membrane, and, in the absence of provisions for cleaning and disposal, the process results in the “messy” spillage of intracellular contents in the extracellular environment. Necrosis can result from a large array of acute injuries and extreme stresses, such as exposure to drastic changes in temperatures or pH, radiation, toxins, osmotic shock, pressure, shear force, etc. There is a trending set of data in support of considering necrosis not as an accidental non-physiological type of cell death but as a molecularly defined and normal process [22,23]. Furthermore, as a variant on the theme of the above-mentioned primary necrosis, a so-called secondary necrosis can become, under some circumstances, the fate of cells dying by other death processes such as apoptosis [24,25] (Figure 1). Indeed, when a terminally apoptotic cell is not cleared adequately, leakage of its intracellular content can trigger an inflammatory response and secondary necrosis [26,27]. In fact, the same event can either lead to apoptosis or necrosis. For instance, lysosomal disruption results in the release of cathepsin proteases into the cytosol and cellular autodigestion. Depending on the nature of the substrate of these cathepsins, what ensues is either apoptosis or necrosis [28]. Other instances of regulated cell death also elicit inflammation. These include necroptosis and pyroptosis, induced by pathogen infection. Necroptosis is a type of regulated necrotic cell death that, unlike accidental necrotic cell death, can be prevented by caspase inhibitors [29,30,31]. In this, necroptosis resembles caspase-dependent apoptosis. Necroptotic cell death is characterized by the formation of cytoplasmic membrane pore complexes through which damage-associated molecular patterns (DAMPs) are released [32]. Terminal morphological events are like those seen in accidental necrosis and include organellar swelling, cell membrane rupture, and the complete collapse of both cytoplasm and nucleus. Because it results in the release of cellular content and inflammation [33,34,35], it is understandable why this process constitutes an important defense mechanism, allowing the self-destruction of the infected cells and preventing further pathogen propagation by attracting attention from the immune cells [36]. Also, since the process is triggered by the inhibition of the caspase machinery by viral proteins and does not necessitate executioner caspases to proceed, this caspase independence, thus, sets necroptosis apart from apoptosis, although both are regulated cell death processes. Other than necroptosis, pyroptosis is also characterized by terminal necrosis-like events that trigger inflammation. Pyroptosis is a programmed necrotic cell death mechanism, specifically dependent upon cleavage of gasdermin D (GSDMD), the designated pyroptosis executioner [37], by inflammasomes and inflammatory caspases 1 and 11/4/5 [38,39,40,41]. In this, it differs from apoptosis, which uses caspases 2, 3, and 6–10. The name was coined from the word “pyro” meaning “fire” or “fever”, the fire of inflammation [39]. Pyroptosis, like necropsis, is a defense mechanism against pathogens [42,43,44]. GSDMD is responsible for forming pores in the cytoplasmic membrane. Indeed, caspases cleave off their N-terminal domain, which opens large pores in the membrane, resulting in swelling and osmotic lysis [45,46,47,48].

Phenotypically, pyroptotic cells are swollen and their membrane is ruptured, causing the release of cellular contents; their chromatin is condensed, and mitochondria lose their membrane potential and leak material [49].

**Figure 2:**
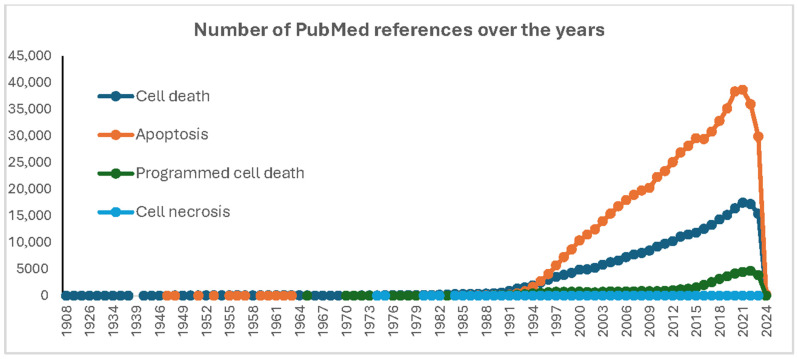
Yearly evolution of publication numbers using four research queries related to cell death, as collated from the NCBI PubMed database.

Although mentions of a programmed type of cell death had appeared earlier [1,50,51], the term apoptosis, coined from the etymology “*apo*” meaning “from” and “*ptosis*” meaning “falling off”, symbolized by leaves falling off trees, was first used in 1972, as a result of a collaborative work between Drs. Kerr, Wyllie, and Currie [52]. The essential distinction here between necrosis and apoptosis is that the latter is “self-inflicted” death. This and similar anthropomorphic expressions such as “cell suicide” dominated the field for the following decades due to the findings comforting the “programmed” nature of the underlying mechanisms. Indeed, decades after its formal recognition, apoptosis remains an active area of research [53], and it actually accounts for the large majority of publications in the area of cell death (Figure 2). Apoptosis, programmed cell death (PCD) or regulated cell death (RCD), is a genetically controlled process that shows a remarkable temporally and spatially regulated sequence of events, as initially showed, for instance, in the development of the nematode *Caenorhabditis elegans* [54], the fruit fly *Drosophila melanogaster* [55,56], *and mice* [57,58,59]. Morphologically, apoptosis is recognizable by the presence of nuclear condensation, membrane blebbing, cell shrinkage, fragmentation, and the formation of the so-called apoptotic bodies. Therefore, while necrotic cell death releases cellular content which elicits an inflammatory reaction, the way apoptotic cellular remnants are disposed of helps escape inflammation by packaging the debris into membranous fragments that are recognized and phagocytosed [60,61,62].

Considering today’s knowledge, the discovery of differences between cell death types should be viewed as important steps in the history of understanding the process rather than defining clear-cut categories. In fact, accumulating evidence indicates that cell death consists of a continuum of morphological and biochemical events [63,64,65]. Many more other ways for a cell to die have been described, including necroptosis, ferroptosis, pyroptosis, alkaliptosis, efferocytosis, parthanatos, autosis, entotic cell death, NETotic cell death, lysosome-dependent cell death, autophagy-dependent cell death, Mitochondrial permeability transition (MPT)-driven necrosis, immunogenic cell death, cellular senescence, mitotic catastrophe, mitotic death [63,66], and more. It would be a daunting task to overview all these types and sub-types of cell death mechanisms. However, one type, autophagy-dependent cell death, deserves special emphasis, if any, in view of the phenomenal interest it has raised in the last few decades (Figure 2). From our viewing angle, it would also be impossible to talk about autophagic cell death without referring to the so-called lysosomal cell death.

Here, again, autophagy has been known for a long time. The term “autophagy” itself, from “*auto*” meaning “self” and “*phagos*” meaning “eat” (self-eating), dates back to the 1860s, but it is the discovery of the lysosome almost a century later that gave it its current biological significance [67,68], i.e., a catabolic process of lysosome/vacuole degradation of intracellular materials to produce nutrients and energy [69,70,71]. Ultrastructural observations provide the most recognizable changes, such as the increase in the number and size of autophagic vacuoles, the engulfment of mitochondria and other organelles as well as of intracellular membranes and cytoplasmic materials to be degraded upon the fusion of the autophagosomes with the lysosomes. Therefore, autophagy has always been regarded as a metabolic adaptative mechanism that allows cells to survive cell death-inducing stimuli [72,73,74]. In the presence of autophagy, cell death can occur in different forms, prominently apoptosis, or in a completely independent manner making use of the autophagy machinery [75]. While the final executive stages can interplay with the apoptotic machinery, initial events are the most distinctive aspects of the process. Although a distinctive aspect of autophagy-dependent cell death is its impact on intracellular membranes, it is ultimately its role in lysosomal degradation and the resulting changes to the metabolic and energetic equilibrium of the cell that make a difference. While the autophagosomal function does not necessarily mean the activation of apoptosis but could simply indicate a normal metabolic process [76], when unbridled, this appetite for its own components is what ultimately causes the demise of the cell. The site of the sentencing in autophagic cell death is the lysosome [77]. These organelles contain a large variety of hydrolases, mainly cathepsins, to degrade proteins as well as phospholipases to degrade phospholipids, among others.

Hydrolases, under an acidic pH, initiate a degradative process that helps recycle the cargo content. Some stress stimuli can result in the perforation of lysosomal membranes and result in lysosomal membrane permeabilization (LMP) [17,78]. In the early stages of an injury, any limited damage can be reversed thanks to the endosomal sorting complex required for the transport (ESCRT) machinery [79,80]. However, when the integrity of the lysosome is compromised and the harm done to the membrane is beyond repair, then ensues lysophagy, a process of selective autophagy-mediated lysosomal clearance. This level of damage to the lysosomes is instrumental in cell death because it results in a wide rupture and massive leakage of content. Keeping in mind that lysosomes are the “stomachs”/“recycling stations” of the cell, any spills would result in the release of both “digestive” materials such as cathepsins and also useless and harmful components and byproducts (e.g., reactive oxygen species). The released material subsequently initiates either apoptotic or necrotic cell death [17,81,82,83,84]. For instance, ROS release, by causing lipid and protein peroxidation and by damaging the lysosomal membrane, results in LMP and lysosomal cell death [77,84,85].

In addition to—and sometimes regardless of—caspases, other cysteine proteases such as calpains and cathepsins, whether aspartic or cysteine, define the executive arm of cell death [86,87]. However, key events in RCD can be executed independently from caspases. For instance, we have seen earlier that caspase-independent apoptosis can be due to the release of lysosomal cathepsins. In addition, while caspase-activated nucleases such as DFF40/CAD (40K DNA fragmentation factor or caspase-activated deoxyribonuclease) mediate apoptotic DNA degradation [88,89], in the absence of a role for caspases, chromatin condensation and chromosomal DNA fragmentation can be mediated by either the apoptosis-inducing factor (AIF) [90] or endonuclease G [91], both nucleases, upon release from the intermembrane space of the mitochondria into the cytosol.

Death by apoptosis involves massive structural dismantlement and decay of the cell, using a “death by a thousand cuts” strategy [92], which is executed by a large number of events of caspase-mediated proteolytic degradation of proteins and nuclease-mediated cleavage of nuclear DNA [93,94,95,96,97]. A large number of proteins are subject to proteolysis (c.f. caspase substrate database of caspase substrates), leading to a variety of phenotypical manifestations [98]. For instance, proteolytic cleavage of nuclear lamins, which form a filamentous nuclear cytoskeleton [99] while also having multiple non-structural functions [100], contributes to nuclear fragmentation [101,102]. Similarly, cleavage of proteins that are important for maintaining the Golgi structure, such as the Golgi matrix protein GM130 [103] or its receptor, the Golgi-stacking protein GRASP65 [104], are key events associated with Golgi fragmentation during apoptosis.

Along with cell shrinkage, apoptosis is also characterized by drastic structural and biochemical changes to the nucleus, most prominently, chromatin condensation, DNA fragmentation, degradation of the nuclear matrix and lamina, and, ultimately, nuclear fragmentation into dense bodies protected by parts of the nuclear envelope [105,106]. A complex sequence of proteolytic events mediated by caspases results in the disruption and dismantling of connections between different nuclear components and compartments, such as the detachment of nuclear membranes from the lamina fibrillar network or from condensed chromatin [107]. It is interesting that disassembly of the nuclear envelope also occurs during mitosis. However, while this process is reversible during mitosis, it is irreversible during apoptosis [107,108,109,110].

In addition to the fragmentation of the nucleus, other organelles are subject to drastic changes, including fission of the mitochondria [111,112], fragmentation of the Golgi [113,114], endoplasmic reticulum (ER) stress [115], and, of course and most spectacularly, fragmentation of the nucleus itself [105]. Dramatic fragmentation is controlled by the fusion/fission machinery and is indicative of mitochondria dysfunction [116,117]. However, the functional significance of mitochondria fragmentation that follows mitochondrial outer membrane permeabilization (MOMP) is controversial [118,119]. While MOMP is a hallmark of apoptosis, it does not always irreversibly lead to apoptosis. Also, mitochondrial membrane remodeling occurs after MOMP independently from caspase activation [120]. Furthermore, limited MOMP has been shown to trigger limited caspase activation, insufficient to trigger cell death but resulting in DNA damage and genomic instability, which contribute to cellular transformation and tumorigenesis [14].

In addition to organelles’ degradation, drastic changes also happen to the overall shape and structure of the cells, including disorganization and dissolution of the cytoskeleton and loss of volume and retraction of the cytoskeleton from the plasma membrane, all leading to shrinkage, blebbing, and the formation of apoptotic bodies [121,122,123,124,125,126,127].

In conclusion, terminal stages of cell death appear to come down to two fundamental processes, either extensive degradation and fragmentation, as seen in apoptosis, or cell lysis and leakage of content, as seen in different types of necrotic cell death (Figure 2).

## 2. Cell–Cell Communication: Dying Alone or Together

The importance of “sticking together” in the response of cells to external stress and injury can be seen even in organisms endowed with much less complex social life than mammals. *Dictyostelium discoideum* are unicellular organisms capable of aggregating into multicellular forms. Indeed, the threat of death by starvation pushes Dictyostelium cells to adhere to each other, forming a multicellular aggregate capable of migration in search of better conditions, eventually saving the majority in the form of spores, while a minority only dies [128]. This dependence of survival upon community life is testament to the importance of intercellular communication for cell life and death [129]. An important consequence of cell death is the dismantling of cell–cell contacts. Apoptosis can be induced by the inhibition of intercellular contact [130]. Reciprocally, intercellular modes can propagate cell death signals.

Cell–cell communication can be realized by many different modes. In addition to secreted chemicals such as growth factors, cells can interact via direct contact between their cytoplasmic membranes. All these modes have been implicated, in one way or another, either in responding to or conveying cell death signals. We have previously reviewed the role of intercellular communication proteins Ephs and ephrins in cell death [129]. Gap junctions and component proteins connexins and pannexins show evident yet complex functions in cell death [131,132,133]. Even the latest to join the family, including extracellular vesicles (EVs) and tunneling nanotubes (TNTs), are part of the cell communication–cell death nexus [134,135,136,137,138,139,140,141].

In the following examples, we will illustrate the complex interplay between cell death and cell–cell communication in three different settings: (1) the establishment of the rules of interplay during some key steps of early development, (2) the interplay in a pathological condition, i.e., during tumorigenesis, and (3) the role of cell–cell communication in the propagation of a cell death message and its use in therapy.

### 2.1. Cell Death and Group Dynamics in Early Development

Even in the absence of external stimuli, cells autonomously undergo selective cell death as part of developmental programs. In fact, billions of cells die daily to ensure proper embryonic development or physiological system function, and, sometimes, extensive cell death can be a sign of pathology and injury [142,143]. These large-scale group deaths, when necrotic, constitute an aggression that, via release of danger-associated molecular patterns (DAMPs), trigger an inflammatory and immune response. However, when part of a normal physiological condition, apoptotic deaths in large groups of cells sculpt organs, help establish multicellular networks, and ensure normal cell turnover, repair, regeneration, aging, etc. [142,144,145,146,147,148,149]. All of this happens to select cells within a community of cells in a precise and programmed manner. The question of how the various modes of cell–cell communication respond to or interfere with this selective cell death program is still widely open.

But let us go back a little in developmental time. Up until the early stages of mammalian preimplantation development, only limited structured cell–cell contacts are formed between the embryo’s cells. This is followed soon after by the establishment of different types of junctions, mainly gap junctions (GJs), adherens junctions (AJs), desmosomes, and tight junctions (TJs), which ultimately lead to the tightening of cell–cell contacts, increase in molecular exchange capabilities, and, hence, metabolic coupling [150,151,152]. The resulting metabolic homogeneity soon clears the way for heterogeneity, following ill-understood mechanisms [153,154]. In the embryo, there is an inverse relationship between metabolism and viability. According to the so-called “quiet embryo hypothesis”, embryos with a less active metabolism have a higher capacity to survive and develop because that relative inactivity generates less lethal damages [155,156,157]. In that metabolically quiet environment, under external stresses, many normal processes such as DNA damage and repair have the potential to trigger death. The body-to-be witnesses its first cell death event when it has only few cells [158,159,160]. As cells divide, the death process is accentuated in the far more multicellular and more differentiated blastocyst mainly through a wave of apoptosis in the inner cell mass (ICM), the part of the blastocyst which gives the fetus [161]. In fact, data suggest that cell death is correlated with cell number and proliferation [161]. It is worth noting that, before the establishment of membrane junctions, the first communication mode to regulate cell death is the paracrine effects of survival growth factors [160]. Also, it seems that the first cell death mode to enter in action is apoptosis. At that stage, when blastocyst cells are sensitive to apoptosis, the fate of cells that have developed gap junction intercellular communication (GJIC) becomes associated with that of their neighbors. It is suggested that GJIC is not necessary for early embryo development and that apoptosis eliminates unwanted and damaged cells before the appearance of the homogenizing action of GJIC [153].

In a metabolism-driven context, it is only normal that autophagy appears in the picture. In fact, it does so in parallel to apoptosis, as early as during gametogenesis, fertilization, and embryo preimplantation [162]. Oocytes in prepubertal rats were shown to be eliminated by the process-sharing features of apoptosis and autophagy while lacking the formation of blebs and apoptotic bodies [163]. During and shortly after egg fertilization, autophagy is responsible for the degradation of maternally inherited proteins in oocytes and for eliminating paternal mitochondrial DNA via mitophagy, thus paving the way for the synthesis of new zygotic proteins and as a prerequisite to preimplantation development [164,165,166,167,168,169]. Throughout early embryogenesis, autophagy occurs in waves where it is activated on and off. The reasons for this are not clear enough, but data suggest a role in assisting cells to dispose of the phagocytosed remnants of apoptotic cells [170,171]. The association between autophagy and cell death is further demonstrated by the fact that embryos deficient in many autophagy genes show massive cell death [172,173]. In mice at three months of age deficient for the autophagy gene Atg5 (autophagy-related 5), specifically in neural cells, granular cells undergo apoptosis. This is due to the loss of synaptic connectivity with Purkinje cells whose axons have swollen [174]. Similar results were found in mice where the autophagic FIP200 gene has been deleted [175].

Later, during early development, migratory neural crest cells are stem cells that contribute to the embryonic development of many tissues and organs in vertebrates [176,177]. Since neural crest cells are organized in sheets or streams which move in multicellular groups [178], it is essential for them to maintain continuous local and long-range communication with each other [179]. This occurs via various modes, including GJIC [180,181,182,183], EVs [184], AJs [185,186], TJs [187,188,189], and Eph/ephrins [190]. However, so far, available data regarding the role of these communication modes in neural crest apoptosis only concern GJs. Gap junctions are essential for the functional coupling and migration of neural crest cells [182]. Early-migrating rat neural crest cells form functional GJ whose pharmacological inhibition results in these cells undergoing apoptosis [191]. In view of the importance of metabolic regulation in neural crests [192], it is expected that autophagy plays an essential role. This is supported, for instance, by the finding that autophagy regulates the production of neural crest cells in early chick embryos [193]. Also, autophagy activated by high glucose levels leads to neural crest apoptosis [194].

### 2.2. Cell Death versus Cell Communication

#### 2.2.1. Extruding the Dead

The intestinal epithelium is among the most rapidly renewing tissues in the body. Stem cells are responsible for replenishing the epithelium in cells, as their division generates precursor cells which proliferate and differentiate, while moving away from the crypt base towards the villus tip, from where they are later shed into the lumen [195,196]. This migratory process, therefore, determines the intestinal epithelial cells’ (IECs) lifespan, and any abnormalities could have serious implications, whether in chronic inflammation-associated diseases or in tumorigenesis [197]. It has been suggested that, in the confinement of the villus tip, the high cellular density resulting from continuous proliferation induces cell death and shedding [198]. This homeostatic process is complex and involves multiple players [199,200]. Dying cells communicate with their neighbors, after which the latter undergo actin/myosin contraction to extrude out the dying cell [201]. The epithelium’s barrier function, measured by electrical resistance, is maintained during the extrusion process and so are the plasma membrane integrity, AJs, and TJs [198]. These junctions are indeed essential for maintaining the intestinal epithelial barrier during IEC shedding [202].

TJs and AJs are adhesive molecular complexes involved in intercellular communication and in the maintenance of normal cellular integrity and cell–cell barriers. TJs are essential for cell polarity in epithelial cells and paracellular permeability in endothelial tissues [203]. Loss of TJ is incriminated in many aspects of tumor progression, including polarity, differentiation, adhesion, migration, invasion, and metastasis [204,205,206,207]. TJs bind to the cytoskeleton and have an intracellular signaling function [203], mediated by an interaction with cytoplasmic adaptor proteins (e.g., zonula occludens, i.e., ZO) or transmembrane linker proteins (e.g., occludin, claudins, and junctional adhesion molecules, i.e., JAMs) [206,208,209,210,211,212,213]. AJs are also important for epithelial tissues and use cadherins and related proteins to connect cytoskeletal structures between cells. Through their cytoplasmic domains, cadherins associate with actin filaments-based cytoskeletal structures [214,215]. AJs link the actin filaments of interacting cells at sites of cell–cell adhesion [216]. TJ rearrangement is important for the process of extrusion of dying cells from the epithelium and barrier maintenance [217]. Although not formally addressed, the fact that IECs’ death involves both a cytoskeletal reorganization and the maintenance of junctional integrity and function directly implicates these junctions in the process of cell death sensing and extrusion of the dying cell [198,201]. Interestingly, the latter communicates out its signal to request extrusion early in the apoptotic process, even before caspase activation and the appearance of apoptotic morphological changes or phagocytosis [201]. If anything, TJs and AJs could ensure the closing off of the intercellular gap left by the extruded cell and maintain confluence within the epithelial barrier [126].

In few words, dying cells take care of their own funerals. In the case of gut homeostasis, this is elegantly accomplished via soliciting the cytoskeletal connection with cell–cell junctions, to extrude them, while leaving behind a preserved epithelial barrier integrity.

#### 2.2.2. Setting Fire

One cannot talk about intestinal epithelium homeostasis without talking about inflammation. In fact, the gastrointestinal tract is a place where cell death, intercellular communication, and inflammation are intimately linked.

The biggest threat to the integrity of the modes of cell–cell communication does not come from within the epithelium. Enteric bacterial pathogens have tremendous effects on junction barriers tasked with keeping them at bay. They try to alter these junctions in order to increase epithelial permeability and, whenever possible, to cross the barrier altogether [218]. In turn, epithelial cell turnover can be increased as a mechanism of expelling pathogens [219]. Such interactions between pathogens and the intestinal epithelium are under the control of an inflammatory process that, when interrupted, could lead to higher levels of apoptosis of IECs, the impaired production of antimicrobial peptides, and, ultimately, the invasion of the mucosa by pathogens [220].

Inflammatory cytokines released by immune cells constitute a threat to the intestinal barrier’s integrity, particularly in conditions such as those found in inflammatory bowel disease [221]. However, this effect of inflammation on increased permeability, in many instances, does not involve apoptosis [222]. In fact, as we have seen above, apoptotic cells signal to their neighbors to extrude them and rearrange TJs to close the gaps [201,202].

Cell death of IECs has been reported to occur not only via apoptosis but also through inflammation-regulated necrosis, necroptosis, and pyroptosis [223]. Excessive apoptosis has been associated with inflammation in the intestine [224]. While this can result from a pathological condition, inflammation in IECs is also part of a mechanism of host protection against potentially harmful enteric pathogens. Another mechanism through which apoptosis could promote inflammation and immunity is by releasing EVs [225,226]. Many mechanisms are at play. For instance, EVs can participate in antigen presentation [226,227]. In addition, extruded apoptotic cells release ABs that, upon engulfment, stimulate cytokine production by the phagocyting cell [228]. Necroptosis has also been involved in intestinal inflammation and tumorigenesis [229,230]. Pyroptosis is also an important part of the host defense arsenal in epithelial tissues [231]. Activation of the inflammasome has been involved in IEC extrusion, along with a loss of plasma membrane integrity and death by pyroptosis. Here, again, the expulsion process is associated with major actin rearrangement in neighboring cells, tasked with maintaining the intestinal epithelium’s integrity [232]. Gasdermins (GSDMs), the principal effectors of pyroptosis [39,41,63,233] which also regulate mitochondrial oxidative stress [234] and are involved in the regulation of autophagy [235,236], have been assigned multiple functions in gut inflammation [237]. While autophagy is important for intestinal epithelium homeostasis [238] and, in fact, for the host’s defense against pathogens [239], it counteracts inflammation [240,241], a function which is important in inflammatory pathogenesis such as in inflammatory bowel diseases [242].

In conclusion, the regulation of the epithelial barrier’s integrity, performed by TJs and AJs, involves a mechanism of extrusion of the dying cells. Pathogens constitute a threat to this process, and it is only normal that, in the response of the host gut to this challenge, inflammatory processes dominate. Therefore, the recourse of the intestinal epithelium to pyroptosis and other inflammation-dependent forms of cell death makes the most sense, even if this entails a risk of perturbing cell–cell interactions and damaging the epithelial barrier’s integrity.

#### 2.2.3. Trying to Fit In

In the intestine, two systems of intercellular communication work in concert to accompany the course of cell renewal, migration, and differentiation. While TJs and AJs are important for the integrity of the epithelial layer, the Wnt/β-catenin/Tcf pathway regulates cell positioning in the crypt in addition to coordinating intestinal stem cells’ migration and proliferation, via Ephs and ephrins [243,244]. And, while the junctional mode has a critical impact on inflammatory diseases, research has proven a tremendous impact of Ephs/ephrins on tumorigenesis.

Ephs/ephrins constitute, in fact, the largest subfamily of receptor tyrosine kinases (RTKs), a fact which fueled interest in using them as targets in cancer therapy [245,246,247]. Ephs and ephrins were initially described as guidance molecules, although it is their discovery in cancer cells which gave them their name (Erythropoietin-producing human hepatocellular carcinoma). This is a large family that enlists both receptors and their ligands called ephrins. In mammals, there are fourteen Eph receptors interacting with eight Ephrin ligands. Both Eph receptors and ephrin ligands are membrane-bound, thus eliciting a bidirectional signaling in the interacting pair of cells [248]. They constitute an intercellular communication mode with many functions, such as the formation of spatial boundaries during normal development [3,4,5,6,7,8,9,10,11,12,13,14,15,16,17,18,19,20]. While they regulate processes such as proliferation, cell death, and invasion, it is their role in cell sorting and positioning that made their fame [249,250], for instance, in regulating the positioning of intestinal epithelial cells within the stem cell niche [244] and coordinating between intercellular communication, migration, and cell positioning [243].

EphB2 and EphB3, two members of the Eph/ephrin family, are major elements of the genetic module that controls the compartmentalization of epithelial cells along the crypt axis and are involved in the regulation of their ordered migration [243]. 

In recent years, a role for Ephs and Ephrins deregulations has emerged in cancer progression and metastasis [251,252,253]. The EphB2 receptor has been reported as a tumor suppressor in the colon [254]. The initial observation was that EphBs are transcriptionally regulated by the β-catenin/Tcf signaling pathway, a major player in human colorectal cancer progression [255,256]. While cells in early lesions of dysplastic crypts and small adenomas, like normal crypt progenitor cells, were found to express EphB2, high-grade tumor areas contained EphB2-negative cells [254,255,256]. Therefore, colon cancer progression is associated with a loss of EphB2 expression. This was further confirmed in the Apc^Min/+^ mouse model of hereditary colon cancer and in many sporadic cancers [257]. Colorectal neoplasms in the Apc^Min/+^ mice usually fail to cross the adenoma-carcinoma transition. Crossing Apc^Min/+^ mice with animals expressing a dominant negative form of EphB2 resulted in a loss of EphB activity that accelerated colorectal tumor progression. In addition, high levels of EphB2 expression were found to be associated with a longer mean duration of survival in colorectal cancer [258], and a loss of EphB2 expression has been reported in colorectal tumors [259,260]. These findings support a tumor suppressor function of EphB2.

Tumor suppression can be achieved by inhibiting cell proliferation or inducing cell death or differentiation. Many Eph/ephrin family members have functions in cell death [129]. EphB2 has been found to regulate the proliferation of intestinal stem cells [244]. Furthermore, EphBs’ role in tumor suppression is performed by limiting the expansion of colorectal cancer cells to specific compartments, rendering difficult the incursion of EphB-expressing cells into areas of high repulsive forces from normal ephrinB-expressing intestinal cells [255]. In fact, a decreasing gradient of EphB2 expression, from the bottom to the top, is found in the proliferative crypt of the small intestine. In the large intestine, EphB2 is expressed only in the progenitor cells at the crypt base. By contrast, the EphB ligands EphrinsB1 and B2 are expressed, also as a gradient, by the differentiated surface and villus cells [255]. During tumorigenesis, the loss of EphB receptors relieves cells from spatial restriction and allows cells to intermingle freely and invade [261]. The tumor suppression effect of EphBs, by controlling the compartmentalization of tumor cells, depends on E-cadherin-mediated adhesion [261]. EphB/ephrinB interaction has also been shown to promote mesenchymal–epithelial transition (MET), reorganizing the cytoskeleton and restoring epithelial E-cadherin/ZO-1-based cell–cell junctions [262]. These effects were also accompanied by the induction of apoptosis [262].

Autophagy is important in intestinal biology through controlling the TJs barrier function, as a survival mechanism, and by regulating intestinal stem cells’ metabolism [263]. Although the data on the function of Ephs/ephrins in autophagy are scarce, the available information supports a direct connection. We have previously shown that EphB2 regulates an autophagy-dependent cell death [264,265]. EphB/ephrinB interaction was shown to use autophagy to clear IECs of intracellular pathogens [266]. Silencing of EphA1 and EphB2 was shown to block autophagy and cell death in colorectal cancer cells [267]. Further investigation is needed.

And now, a few words about the function of Ephs and ephrins in inflammation and immunity that has increasingly been acknowledged [268,269]. The role of these proteins in angiogenesis is one of the first and most studied aspects of their biological functions [270]. A major part of the function of Ephs/ephrins in gut inflammation involves synaptic plasticity and the neuroimmune regulation of intestinal inflammation-related pathogenesis [268]. The relevance of the functions of Ephs/ephrins in inflammation and immunity in colorectal cancer remains, however, to be further examined. A possibility is via a role in the tumor’s immune microenvironment [246,271]. However, how cell death could be involved is an important question deserving of attention in view of the above-discussed association between inflammation and cell death.

#### 2.2.4. Surviving Attacks

Since intercellular communication is functionally connected to cell death, it is no surprise that it plays a role in cancer therapeutic resistance. However, the extent to which the balance between the two processes, i.e., communication and death, is important for drug resistance, could be easily identified as an area of unmet need in cancer research.

Data regarding the role of Ephs and ephrins in colon cancer drug resistance is still sporadic. EphB2 was found as one of the genes whose expression is increased in colon cancer cells resistant to platinum or taxane [272]. Acquisition of resistance to cetuximab, an antibody-based EGFR inhibitor, is associated with an increase in EphB3 expression levels and EphB3/EGFR binding in colorectal cancer cells. The mechanism of resistance can be overcome by inhibiting EphB3 expression, which allows cetuximab-induced apoptosis [273]. Claudin-1 (CLDN1) promotes colorectal cancer (CRC) cells’ chemoresistance by interacting with and stabilizing EphA2. This results in enhancing the antiapoptotic AKT signaling pathway and promoting cancer stemness [274]. Directly targeting EphA2 with a tyrosine kinase inhibitor decreases cell proliferation and induces cell death in CRC cells [275,276,277]. Based on the finding that, in response to DNA damage, the ephrinB2-encoding gene is a transcriptional target of p53, a key apoptosis regulatory protein, knock down of ephrinB2 expression was used to restore apoptosis and 5-FU chemosensitivity in mutant p53-harboring CRC tumors [278]. The physical and functional association between EphA2 and EGFR plays a role in the resistance to EGFR-targeting therapies [279]. High EphA2 expression levels are associated with a worse outcome in patients treated with cetuximab [280]. Metastatic CRC cells that have NRAS (neuroblastoma RAS viral oncogene homolog)-activating mutations are resistant to cetuximab. A failure of cetuximab to downregulate EphA2 expression in NRAS-mutant CRC cells, in comparison with cetuximab-responsive CRC cells, was suggested as a potential contributor to resistance to this drug [281]. The use of the ephrinA1 ligand to activate EphA2 succeeds in restoring cetuximab activity in NRAS-mutant colorectal cells [281]. Although it is not clear whether the mechanism involves an effect on apoptosis or another type of cell death, it was shown to involve the suppression of mitogen-activated protein kinase (MAPK) and AKT hyperactivation. The roles of these pathways in both apoptosis and Ephs/ephrins signaling are established [282]. An EphA2 small-molecule inhibitor could be used successfully to reverse cetuximab resistance in CRC cells by inducing apoptosis and cell cycle G1–G2 arrest and inhibiting the MAPK and AKT pathways [283]. Although shown in a different type of cancers, i.e., head and neck squamous cell carcinoma, resistance to cetuximab and radiotherapy (RT) was associated with elevated EphB4 and ephrinB2 expression levels [284]. The therapeutic response to cetuximab-RT could be improved by concomitantly blocking the EphB4–ephrinB2 interaction that results in the inactivation of the AKT and MAPK pathways, a decrease in proliferation, and an increase in apoptosis [284]. Similarly, in non-small-cell lung cancers, silencing of ephrinB3 sensitizes cells to a combined treatment with the kinase inhibitor PKC 412 and ionizing radiation [285]. Here, again, the MAPKs and Akt signaling pathway are involved, which elicit a decreased proliferation, increased apoptosis, mitotic catastrophe, and senescence signaling [285]. These data point to a prominent role for the MAPK and AKT pathways and apoptosis. However, the link with Eph/ephrin-mediated intercellular communication awaits further direct investigation. Interestingly, MAPK inhibition along with tyrosine phosphorylation of cell junction proteins such as CLDN1 was observed in cetuximab-responsive tumors compared to their resistant counterparts [286].

While apoptosis’ prominent role in mechanisms of CRC drug resistance is well documented [287,288,289], data are in favor of an equally critical function for autophagy and autophagy-dependent cell death [290,291]. A balance between apoptosis and autophagy, controlled by p38MAPK, has an important role in resistance to 5-FU [292]. Autophagy is involved in the resistance of CRC cells to EGFR targeting using a monoclonal antibody [293]. In this case, autophagy acts as an adaptive survival mechanism because, when inhibited, the response to the drug by increasing cell death is improved using an autophagy inhibitor [293]. In fact, a combined targeting of both EGFR and autophagy is a tempting yet still underexplored strategy to bypass drug resistance in CRC [294]. To date, only a handful of publications have addressed the regulation of autophagy by Ephs and ephrins in cancer, including in CRC cells [264,265,266,275,295]. Whether this function could impact therapeutic drug responsiveness is unknown. There are even less data regarding other modes of cell death, especially ferroptosis or pyroptosis. Ferroptosis is increasingly viewed as a possible therapeutic target in CRC [296,297]. When other modes of cell death such as apoptosis fail, the induction of ferroptosis succeeds in eliminating CRC cells [298]. This is understandable, knowing that inhibition of ferroptosis by iron sequestration results in CRC progression and resistance to 5-FU [299].

In addition to Ephs and ephrins, the role of connexins in drug resistance has also been recognized, although it is sometimes paradoxical depending on whether they act via GJIC or not. Expression of some connexins has been associated with chemoresistance in a GJIC-independent manner [300,301,302]. However, GJIC can also directly contribute to these mechanisms, as in the case of fibroblasts which protect lung tumor cells from death by connecting with them via GJICs, thus resulting in cancer cells’ chemoresistance [303]. The BE mediated by GJIC increases cisplatin cytotoxicity, and the inhibition of Cx43 expression results in drug resistance [304]. In fact, the induction of connexin expression and/or the activation of GJIC have long been viewed as strategies to overcome chemoresistance or, as will be seen below, to improve the response to gene or radiation therapies. In CRC, 5-Fluorouracil (5-FU) is one of the most important chemotherapeutic drugs in use. However, resistance emerges to this drug [305]. A correlation exists between a loss of CX43 expression, metastasis, and poor prognosis, while restoring Cx43 expression leads to the suppression of CRC progression and an increase in the sensitivity to 5-fluorouracil (5-FU) [306]. In addition to 5-FU, the resistance of CRC cells to oxaliplatin and irinotecan is also associated with a loss of Cx43 expression [307]. The use of resveratrol, a natural polyphenol, has been explored to chemosensitize CRC cells to 5-FU. The mechanism involves the upregulation of cell–cell communication molecules that constitute desmosomes, gap and tight junctions, and adhesion molecules, while also enhancing the onset of apoptosis [308]. Similarly, one of the mechanisms through which all-trans retinoic acid (ATRA) is able to restore the resistance of CRC cells to paclitaxel is by improving Cx43-dependent GJIC [309]. However, this effect relies more on a role for Cx43 in reducing CRC cells’ migration and invasiveness rather than inducing apoptosis [309]. The connection between these functions of connexins and GJIC in drug resistance with various types of cell death has not been addressed specifically. Some data from other types of cancer could provide some insight. For instance, while Cx43 was reported to have a role in resistance of glioblastoma (GBM) cells to temozolomide (TMZ) thanks to an anti-apoptotic effect [302], when combined with a Cx43 inhibitor, responsiveness to TMZ is improved by inhibiting AKT signaling and inducing both autophagy and apoptosis [310]. Furthermore, under regulation by EGFR/MAPK signaling, Cx43 expression is increased in TMZ-resistant GBM cells [311]. Knocking down Cx43 expression sensitizes GBM cell to TMZ [311]. Perhaps, more spectacularly, through establishing GJIC with neighboring astrocytes, GBM cells can resist TMZ-induced apoptosis, an effect which is prevented by the knockdown of astrocytic Cx43 expression [312]. These and other data put forward the potential use of connexin and GJIC targeting as an approach to counteract TMZ resistance [313]. Connexins and autophagy have a reciprocal relation, each being both target and regulator of the other [314]. This is the case in cervical cancer cells where the overexpression of connexin32 (Cx32) promotes autophagy in a GJ-independent manner, subsequently inducing apoptosis [315]. Nevertheless, within this equation, Cx32 has another GJIC-independent function, as it suppressed apoptosis induced by combined treatment with an autophagy inhibitor and cisplatin [316]. This anti-apoptotic effect is mediated by the Cx32-driven upregulation of EGFR expression and could be abrogated using an EGFR inhibitor [316].

In summary, intercellular communication, whether via Eph/ephrin, EVs, GJs, TJs, or AJs, has essential functions in the homeostatic regeneration of the gut, its response to pathogens, its blockade of tumor progression, and its response to therapeutic agents. While some of these roles directly implicate apoptosis and, to a lesser extent, autophagy, some other roles rely more on a role as positioning, sorting, and guidance systems (Ephs and ephrins).

### 2.3. The “Bystander Effect”

The propagation of cell death signals has long been associated with gap junction intercellular communication (GJIC), a junction-based mode of interaction. GJs are made of intercellular channels, composed of the connexin proteins [317] which allow direct cytoplasmic continuity between cells. This structure allows the passive exchange of a plethora of small molecules, including calcium, ions, nucleotides, cAMP/cGMP, inositol 1,4,5-triphosphate, ATP, ADP, amino acids, and other metabolites and signaling molecules [318,319,320] as well as cellular electrical coupling and synchronization [321,322]. In addition to connexin-based channels, there are hemichannels based on the pannexin proteins that connect between the cytoplasm and the extracellular milieu. Both connexins and pannexins have an important role in cell death [133].

GJIC shows both pro-apoptotic [323,324,325,326,327] and anti-apoptotic [328,329,330,331,332] functions. A well-known consequence of the pro-apoptotic role of GJIC is the so-called “bystander effect” (BE), whereby a cell can transmit a cytotoxic message to its neighbors when it is connected to them via GJs [333,334]. The classical example is the use of the herpes simplex virus-thymidine kinase (HSV-tk)/ganciclovir (GCV) system as a cancer gene therapy approach. The HSV-tk gene product converts the antiviral drug GCV into GCV triphosphate, an analogue with pro-apoptotic effects. Even if only a single cell is targeted with this system, pro-apoptotic signals can be transmitted by the targeted cell, via GJs, and induce cell death in its by-standing neighbors, even if the latter do not express the necessary HSV- gene [335,336].

A similar BE is observed in cells that are exposed to ionizing radiation (IR), a long-known phenomenon with an important impact on human health [337,338,339]. Here, again, the damage inflicted upon a cell population is shared with non-irradiated neighboring cells with which they are in a GJIC [340].

In both examples of BE, the first question is about the nature of the message shared by donor cells with acceptor cells. In the gene therapy case, following the classical pro-drug activation process, GCV triphosphate analogue production is performed in the donor cell and reaches the acceptor cells. GCV triphosphate is a DNA polymerase inhibitor that is incorporated within the newly synthesized DNA, resulting in chain termination and single-strand breaks [341], thus triggering apoptosis. The model for the pro-apoptotic mechanism involves putatively a p53-dependent transcription of the CD95 receptor, which becomes activated in the absence of ligand stimulation, subsequently leading to the recruitment of the extrinsic apoptosis machinery components FADD and caspase-8, triggering the caspase cascade [342]. Similarly, radiation-associated BE involves the induction of different forms of chromosomal and genomic DNA damage, which trigger a “DNA damage response” (DDR), shared by the targeted cell with by-standing neighbors not directly subject to irradiation [343,344,345,346,347]. Once transduced, the DDR signal activates an array of molecular pathways geared towards repairing the damage [348] but eventually activating p53, p63, and p73 and eliciting apoptosis [349,350,351,352]. In addition to nuclear DNA damage, other components from the cytoplasm can be targeted by radiation [353,354], thus resulting in the generation of deleterious molecules which can also be shared by BE. These include DNA synthesis-inhibiting molecules [37] and free radicals generated by stimulated mitochondria, which can cause DNA damage [347,353]. The radiation-induced production of reactive oxygen species (ROS), an important event [355], can increase lysosomal membrane permeability, the release of acid sphingomyelinase, the activation of ceramide synthase, and, ultimately, lead to apoptosis [356,357,358,359,360]. 

BE-associated genotoxicity also triggers non-apoptotic forms of cell death, depending on the nature of the damage [361,362]. In addition, autophagy has been associated with a response to IR and, interfering with it, results in cell sensitization to radiation therapy [363,364,365,366]. Furthermore, irradiation could induce autophagy in both targeted and bystander cells [367] via the transfer of ROS molecules [368]. Autophagy might function as a survival response, ridding the injured cell of damaged cellular components including mitochondria, via mitophagy, a mitochondria-specific form of autophagic degradation. However, multiple data feed the ambiguity concerning the role of autophagy in the response to radiation and the BE. For instance, while autophagy protects cells from genomic instability and DNA damage [369,370], mitophagy increases radiation-induced DNA damage and cell death [371]. In addition to inducing autophagy, IR also induces ferroptosis, a type of iron-dependent and lipid peroxidation-mediated cell death mechanism [372,373]. In fact, ferroptosis is closely associated with autophagy and is, therefore, important for the metabolic machinery of cell death and survival [374,375,376,377]. Ferroptosis, however, relies heavily on lipid metabolism [377,378,379,380]. The effect of IR on ferroptosis is due to its impact on the generation of lipid peroxides and increase in iron metabolism [381]. In return, the induction of ferroptosis has been shown to sensitize cells to radiation [382]. Although different in many ways from apoptosis (absence of chromatin condensation, nuclear fragmentation, and formation of blebs) and necroptosis (no membrane rupturing) [383], ferroptosis is considered a regulated necrotic cell death. Its main morphological feature is the alteration of the mitochondria [373], and the latter’s functions are essential for this process to occur [376,384,385,386]. Although studies formally scrutinizing the link between ferroptosis and BE are lacking, there are many indications in support of this link, the first of which is the importance of mitochondria for both processes. Inhibition of the GJIC protein Cx43 blocks ferroptosis and its associated cell death [387,388]. Interestingly, depletion of the GJ protein pannexin1 attenuates lipid peroxidation and iron accumulation and, subsequently, inhibits ferroptotic cell death [389]. This is not surprising, as pannexins are involved in forming large pores and GJ hemichannels [133,390,391] and are important for signal propagation, particularly electrical coupling [392]. Furthermore, many studies have shown that ferroptosis and the cell death it generates are propagated to neighboring cells [383,393,394,395]. Therefore, BE is not restricted to apoptosis, and, in contexts where ferroptosis is favored over other cell death types, cell death propagation between targeted and non-targeted cell is possible.

In another respect, cells can share mitochondria as part of the BE. These organelles are vital for the cell’s metabolic and energetic functions and, hence, for the cell’s death and survival mechanisms. Intact mitochondria as well as fragments are known to be shared between cells via multiple communication modes, including GJs, TNTs, and EVs [396,397,398,399,400,401,402,403,404]. In doing so, healthy mitochondria can, through a fusion mechanism, restore the function of damaged mitochondria in communicating cells, thus rescuing the cell from apoptosis [136,405]. Metabolic boost by mitochondria transfer between astrocytes and glioblastoma cells increases mitochondrial respiration and the activity of metabolic pathways, thus promoting cell proliferation and tumorigenicity [406]. Cytosolic irradiation results in mitochondrial depolarization [407] and extensive fragmentation [408], launching a cascade of ROS and apoptosis. Since intracellular ROS molecules are mainly generated in the mitochondria, increasing mitochondrial fusion activity can protect cells from irradiation-induced apoptosis [408]. Using a cell co-culture system, it has been shown that healthy mitochondria transfer restores the DNA damage repair response of by-standing irradiated cells [405]. Therefore, it seems that, while irradiated cells use the BE to share cytotoxic signals with neighboring cells, the latter use the BE to send back healthy mitochondria in an attempt to restore damaged organelles and rescue the irradiation-targeted cells from death.

Although initially associated with GJIC, the BE has later been found to involve other modes of cell–cell communication as well. Grouped under the generic name of extracellular vesicles (EVs) is a large and diverse population of membrane-based vesicles released by cells into the extracellular environment [409,410,411,412,413,414,415,416,417,418]. EVs are active members of the intercellular communication toolkit and are involved in the transfer of a large variety of molecules and organelles [419,420,421,422,423,424,425,426,427,428,429,430,431,432]. Most prominent among the EVs are exosomes and apoptotic vesicles. In fact, EVs are released by many cell types in normal, stressed, or pathological conditions [83,433]. Cells exposed to IR respond by releasing exosomes [434], and it has been reported that these EVs are part of radiation-induced BE [435,436,437,438,439,440,441]. EVs appear to be particularly associated with BE signal perpetuation, as BE acceptor cells are also capable of inducing BE in other cells via EVs [437] and also by transferring microRNAs responsible for downregulating the expression of TGFβ1, which triggers BE by increasing intracellular ROS [442,443,444].

As discussed earlier, dying cells release apoptotic bodies and small vesicles, allowing them to communicate with neighboring cells [21,52,83,411,414,445,446,447,448,449,450,451,452,453]. It has been suggested that HSV-tk-targeted dying cells release apoptotic vesicles that, once taken up by nearby non-targeted acceptor cells, also enter apoptosis [454]. EVs are also capable of mitochondria transfer [402,403,404]. 

Irradiated cells rush to build a denser-than-normal network of TNTs [455]. TNTs can mediate the transfer of mitochondria [396,397,398,399,400,401] and are thus capable of reversing radiation-induced apoptosis at its early stages [136].

In conclusion, one of the fundamental contributions of different intercellular communication modes is the amplification of cellular signals. While this has advantages at the tissular level to ensure a level of intercellular coordination and synchronization, it can become detrimental to the cells when it is taken advantage of, as exemplified by the BE cytotoxic effects. The BE has indeed important biological and clinical implications, particularly in disease therapy [346,456,457].

## 3. Concluding Remarks

The question of what constitutes a dead cell has been and remains an open and complex one. For decades after the discovery of necrosis and apoptosis, pharmacological inhibition and genetic loss of function experiments have reached (the intriguingly obvious) conclusion that irreversibility is the defining parameter. Therefore, damages to the mitochondria and possibly some of the executioner caspase-driven proteolytic events would be essential indicators. However, it is now clear that what defines irreversibility is far from being ubiquitous. Cells can survive caspase activation or mitochondrial loss of integrity following a process called anastasis [8]. Interestingly, it has been shown that early recovery from apoptotic stress was associated with entry in a proliferative state followed by migration [15]. These data suggest that the irreversibility of cell death, at least via apoptosis, depends on the ability of cells to engage in a different homeostatic process, e.g., from growth arrest to proliferation and from the latter to migration. Indeed, multicellular organisms have developed a coupling between homeostatic processes that is lacking in less developed organisms such as *Dictyostelium discoideum* [128]. Further investigation of this coupling hypothesis would have an important impact particularly for the understanding of pathological processes whereby getting rid of diseased cells is an objective that can be challenged by recovery from and resistance to cell death.

Although many modes of cell death processes have been described over the years, it appears that they share many executive mechanisms and differ mainly in their morphological features, their triggers, and whether they are strong inducers of an inflammatory response. Another distinction is the way in which specific modes interplay with other homeostatic processes such as proliferation, migration, and differentiation. To these distinctions, we would like to suggest adding how each cell death process interplays with cell–cell communication modes. The role of intercellular communication in propagating cell death signals has been a very promising axis of research, particularly towards developing gene and radiation therapies. While this has mainly focused on the GJIC-mediated bystander effect, understanding the role of other modes of communication could provide a better refinement of therapeutic strategies.

Cell–cell communication is critical for the survival and growth of multicellular organisms. In addition to growth factors and other chemical moieties, different modes of membrane-based interactions exist that are endowed with important functions and that, when defective or lost, can have dramatic impacts on cell life or death and prove instrumental in human diseases such as cancer [129,458]. It goes without saying that, considering that these interactions happen at the gate of cells, we are more concerned here with the initiation phase of cell death rather than its later phases. While there is a variety of modes of cell death, there is also a large variety of cell–cell communication modes. Cell death and intercellular communication are intertwined along the normal developmental stages of organisms and remain so in pathological conditions. On the brink of death or even after death, as a last message to the cellular world, a cell dying by apoptosis uses fragments of its body as a messenger to communicate with neighbors. It can ask to be extruded, as seen in the case of the intestinal epithelium, and, to this end, relies on help from junctional cell–cell communication structures [201]. It sends “find-me” messages to be recognized and “eat-me” signals to be engulfed by phagocytosis [62,459]. Even if it serves the purpose of finalizing cell death itself, the process of phagocytosis can be considered as a last intercellular communication of the dying cell with the cellular world [460]. Phagocytosis is not a mere waste collection system, it is actively involved in the apoptotic cell death process itself [461,462,463], although the mechanisms behind this are unknown. Here, a major difference between the modes of cell death is that, unlike apoptosis, cell death modes that lead to a necrotic outcome will communicate with their microenvironment via inflammation. While many research laboratories are looking into this, the call to action from dying cells towards intercellular communication molecules and structures still deserves greater attention. We hope that the examples used here will convey the importance of this aspect of cell death, linking it to cell communication.

## Data Availability

Not applicable.
